# Integrative genomic and transcriptomic profiling of pulmonary sarcomatoid carcinoma identifies molecular subtypes associated with distinct immune features and clinical outcomes

**DOI:** 10.1002/cai2.112

**Published:** 2024-04-15

**Authors:** Sahil Seth, Runzhe Chen, Yang Liu, Junya Fujimoto, Lingzhi Hong, Alexandre Reuben, Susan Varghese, Carmen Behrens, Tina McDowell, Luisa Solis Soto, Cara Haymaker, Annikka Weissferdt, Neda Kalhor, Jia Wu, Xiuning Le, Natalie I Vokes, Chao Cheng, John V. Heymach, Don L. Gibbons, P. Andrew Futreal, Ignacio I. Wistuba, Humam Kadara, Jianhua Zhang, Cesar Moran, Jianjun Zhang

**Affiliations:** ^1^ Department of Genomic Medicine The University of Texas MD Anderson Cancer Center Houston Texas USA; ^2^ TRACTION The University of Texas MD Anderson Cancer Center Houston Texas USA; ^3^ Graduate School of Biomedical Sciences The University of Texas MD Anderson and the University of Texas Health Science Center Houston Texas USA; ^4^ Department of Thoracic/Head and Neck Medical Oncology The University of Texas MD Anderson Cancer Center Houston Texas USA; ^5^ Department of Translational Molecular Pathology The University of Texas MD Anderson Cancer Center Houston Texas USA; ^6^ Department of Epidemiology The University of Texas MD Anderson Cancer Center Houston Texas USA; ^7^ Department of Pathology The University of Texas MD Anderson Cancer Center Houston Texas USA; ^8^ Department of Imaging Physics The University of Texas MD Anderson Cancer Center Houston Texas USA; ^9^ Department of Medicine Baylor College of Medicine Houston Texas USA

**Keywords:** genomic, immune, pulmonary sarcomatoid carcinoma, survival

## Abstract

**Background:**

Pulmonary sarcomatoid carcinoma (PSC) is a rare and aggressive subtype of non‐small cell lung cancer (NSCLC), characterized by the presence of epithelial and sarcoma‐like components. The molecular and immune landscape of PSC has not been well defined.

**Methods:**

Multiomics profiling of 21 pairs of PSCs with matched normal lung tissues was performed through targeted high‐depth DNA panel, whole‐exome, and RNA sequencing. We describe molecular and immune features that define subgroups of PSC with disparate genomic and immunogenic features as well as distinct clinical outcomes.

**Results:**

In total, 27 canonical cancer gene mutations were identified, with *TP53* the most frequently mutated gene, followed by *KRAS*. Interestingly, most *TP53* and KRAS mutations were earlier genomic events mapped to the trunks of the tumors, suggesting branching evolution in most PSC tumors. We identified two distinct molecular subtypes of PSC, driven primarily by immune infiltration and signaling. The Immune High (IM‐H) subtype was associated with superior survival, highlighting the impact of immune infiltration on the biological and clinical features of localized PSCs.

**Conclusions:**

We provided detailed insight into the mutational landscape of PSC and identified two molecular subtypes associated with prognosis. IM‐H tumors were associated with favorable recurrence‐free survival and overall survival, highlighting the importance of tumor immune infiltration in the biological and clinical features of PSCs.

## Graphical abstract

Overview of clinical characteristics of pulmonary sarcomatoid carcinoma (PSC) cohort and exemplary histopathologic images of three different types of PSC cancers, including Spinfle cell carcinoma, pleomorphic carcinoma, and giant cell carcinoma. Tumors and adjacent normals were subjected to whole‐exome sequencing (*N* = 18), RNA sequencing (*N* = 17), and comprehensive cancer panel (*N* = 21).

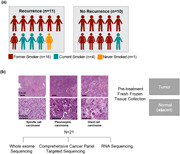

**Abbreviations**
CCP: comprehensive cancer panel
CNV: copy number variation
ICB: immune checkpoint blockade
IM‐H: Immune High
IM‐L: Immune Low
LSCC: lung squamous cell carcinoma
NSCLC: non‐small cell lung cancer
OS: overall survival
PSC: pulmonary sarcomatoid carcinoma
RFS: recurrence‐free survival
RNASeq: RNA sequencing
WES: whole‐exome sequencing

## 1 INTRODUCTION

Pulmonary sarcomatoid carcinoma (PSC) is a rare subtype of non‐small cell lung cancer (NSCLC) characterized by the presence of both epithelial and sarcoma‐like components, accounting for 0.1% to 0.4% of all newly diagnosed lung cancers [1, 2]. The 2021 World Health Organization (WHO) classification defines three different histological subtypes of PSC: pleomorphic, carcinosarcoma, and pulmonary blastoma [3]. Due to the heterogeneity among these tumors, diagnosis is often challenging, especially when only small biopsy specimens are available [4, 5, 6, 7]. PSCs are overall resistant to conventional platinum‐based chemotherapy and are associated with poor prognosis compared with other NSCLC subtypes [8].

Targeted and immune therapies in recent years have greatly advanced the treatment of NSCLC, providing new opportunities for the therapeutic strategies of PSC. Previous studies have shown a high prevalence of *TP53* (60%–74%) [9] and *KRAS* (20%–43%) [4, 5, 10], followed by *PIK3CA, MET, NOTCH, STK11*, and *RB1* [6, 9] in PSCs. *KRAS* mutations were reported to be associated with poor prognosis [4, 5, 10]. *MET* exon 14 skipping mutations have been recently identified in PSC, providing a targeted therapy option for PSC [4, 5, 6]. In addition, PSC was among the tumors with high tumor mutation burden (TMB), which has been reported to be associated with superior response to immune checkpoint blockade (ICB) therapy [1, 8, 11], offering new hope for patients with PSC [12].

The underlying molecular pathophysiology accounting for the sarcomatoid phenotype and its distinct characteristics, as well as potential mechanisms related to the prognosis of PSC, are poorly understood, largely due to the lack of appropriate materials for comprehensive profiling. In this study, we performed multiomics profiling of 21 pairs of PSCs with matched normal lung tissues through targeted high‐depth DNA panel, whole‐exome, and RNA sequencing. We describe molecular and immune features that define subgroups of PSC with disparate genomic and immunogenic features as well as distinct clinical outcomes.

## 2 METHODS

### 2.1 Study population

Tumor and matched (histologically) normal tissue samples were obtained from 21 patients with PSC before treatment. Written informed consent for sample collection and analysis was obtained from all patients. This study was performed in accordance with the Declaration of Helsinki and was approved by the Institutional Review Board at The University of Texas MD Anderson Cancer Center. All patients provided written informed consent.

### 2.2 DNA sequencing

All 21 pairs of tumor‐normal samples were deeply sequenced using Ion Torrent's comprehensive cancer panel (CCP) of 409 cancer genes (mean sequencing depth 355 ± 78). Of these, 18/21 samples with high‐quality DNA were subjected to whole‐exome sequencing (WES) using the Ion Torrent AmpliSeq platform, targeting 195,427 exons across 19,070 genes (median sequencing depth 225 ± 20). Libraries were prepared using the manufacturer's specifications and sequenced using the Ion Proton System.

### 2.3 Mutation calling on DNA sequencing

Raw sequencing reads were aligned using the Torrent Mapping Alignment Program (TMAP‐4.0.6) to HG19 whole‐genome reference. The mark duplicates step was skipped since these data were derived from single‐end sequencing. We used a consensus calling approach on IonTorrent WES data using three different callers (MuTect, Mutect2, and the platform's proprietary method, Ion‐Reporter) to derive the mutational landscape of PSC. We used a pooled normal consisting of 21 adjacent normal tissues to filter out germline variants and sequencing artifacts.

Mutations were called on WES and CCP using Ion‐Reporter caller (based on FreeBayes). A second set of calls was derived following GATK best practices, proceeding with somatic calls using Mutect. In addition, a pooled normal was created using MuTect2 to filter out sequencing artifacts. This was used as a pooled normal for MuTect2 and MuTect. Mutations were annotated using VEP and ICMG tiered criteria. A consensus calling approach was used by combining data from the three callers to arrive at confident somatic mutations. Two filters were established: filter (1) focusing on confident somatic calls in any gene and (2) reasonable variants in known oncogenes and tumor suppressors. Filter 1 required that a mutation (1) “PASSED” by two of three mutation callers (MuTect, MuTect2, or IonReporter) and (2) showed forward and reverse variant allele frequencies >5% to remove strand biases mutations. Filter 2 required that a mutation shows forward and reverse VAF > 5% and either has a TCGA pan‐cancer count >3, curated in clinVar, or established as ICMG tier 1 or tier 2 mutations. We refer to this filtered list as the F1/F2 mutations list, which was used for TMB analysis. In total, the F1/F2 criterion filtered down mutations to *N* = 5147; of these, 28 satisfied both the F1 and F2 criterion, 68 mutations were hotspot/known oncogenic mutations that failed the strict F1 criterion, and 5051 other somatic mutations.

To find pathogenic somatic mutations, we started with *N* = 99 mutations satisfying the F2 criterion and curated them using WES, CCP, and RNASeq bam files in IGV, with *N* = 46 mutations in 28 genes (Supporting Information: Table S1).

We employed a rule‐based criterion using ACMG guidelines [13] to annotate the variants and selected all (1) ACMG tier 1 & tier 2 mutations, or (2) if the specific mutation had a pan‐cancer count >3 or (3) if the mutation was annotated in ClinVar [14], as elaborated in the methods. Using these genes, we distilled for damaging nonsynonymous mutations or stop gain/splicing mutations in cancer genes (Supporting Information: Figure S1A,C).

### 2.4 Copy number variation (CNV) calling pipeline

Data from all matched normal were pooled using GATK CNV [15] to create an improved identification of CNV events from artefactual depth‐ratio variation (*N* = 18). CNV segmentation was performed using TitanCNA [16], followed by tree structure using PhyloWGS [17]. Purity was assessed as the total size of subclones from PhyloWGS, which incorporates CNV events and cancer cell fraction (CCF) of SNVs.

### 2.5 Expression profiling

17/21 samples were used for Ion Torrent‐enabled RNA sequencing (RNASeq). RNA was extracted, and libraries were prepared using Ion Total RNA‐Seq Kit v2 (https://www.thermofisher.com/order/catalog/product/4475936). We used two‐pass star alignment to align sequencing reads to the hg19 human genome and HTSeq to get gene‐level counts using the TCGA V2 GAF file (ref). Counts were normalized for between samples variation using the TMM method of edgeR [18]. Differentially expressed genes (DEGs) between clusters 2 and 1 were calculated using the Fisher exact test. Pathway analysis was performed using GSEA and Enrichr [19, 20]. We manually annotated immune‐related pathways in MSigDB v5.1 Reactome DB, using terms such as “immune,” “TCR,” “Interferon,” “antigen,” and so forth. Immune cell deconvolution was performed using MCPCounter [21], CIBERSORT [22], ConsensusTME [23], and xCell [24].

### 2.6 Statistical Analysis

The statistical analysis and generation of figures were conducted using the R. Box plots and other statistical summaries were plotted using R package ggstatsplot. Pearson's correlations were employed to evaluate the relationship between two continuous variables. When analyzing one nominal and one continuous variable, the Student *t*‐test was utilized for normally distributed data to examine difference between two groups. For non‐normally distributed data, the Wilcoxon signed‐rank test and Mann‐Whitney test were employed for paired and independent comparisons, respectively. Chi‐squared test was employed to compare categorical variables in two groups. Survival analysis was conducted using the log‐rank test, using the R package survminer and survival. Multivariate analysis was performed to determine correlations between multiple factors by analyzing two or more variables simultaneously. *P*‐values less than 0.05 were considered to be statistically significant.

## 3 RESULTS

### 3.1 Clinical characteristics of patients with PSC

**Figure 1:**
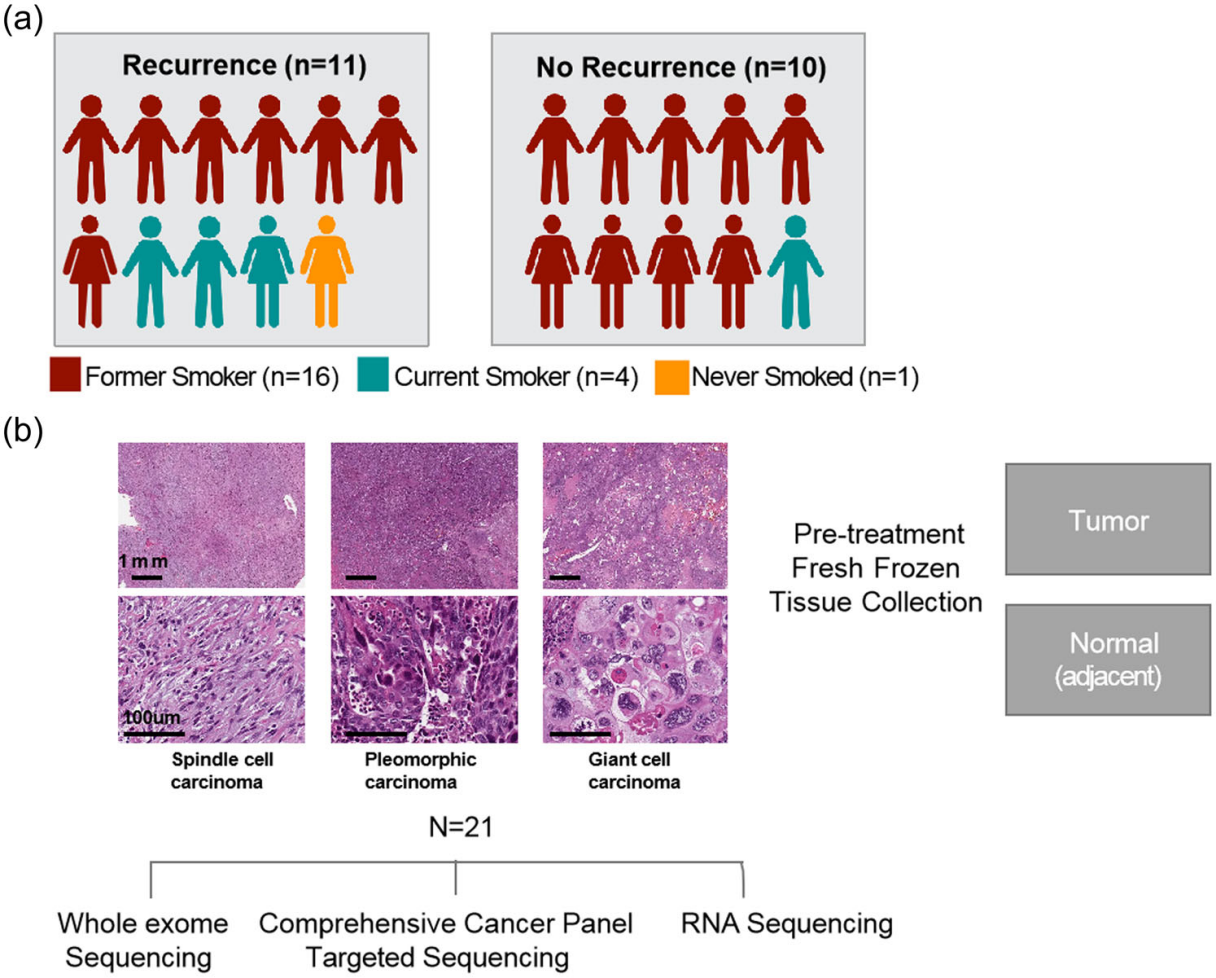
(a) Overview of clinical characteristics of pulmonary sarcomatoid carcinoma (PSC) cohort (*N* = 21) patient tumors, including 11 patients with recurrence and 10 patients with no recurrence. (b) Exemplary histopathologic images of three different types of PSC, including Spinfle cell carcinoma, pleomorphic carcinoma, and giant cell carcinoma. Tumors and adjacent normals were subjected to whole‐exome sequencing (WES) (*N* = 18), RNA Sequencing (*N* = 17), and Comprehensive cancer panel (*N* = 21).

We retrospectively identified 21 patients with the diagnosis of PSC in our institution (Figure 1a). The clinicopathological characteristics of these 21 patients are summarized in Table 1. The median age of the studied cohort was 68 years (range: 49–80). Fourteen (67%) patients were male, and the majority (*N* = 20, 95%) were smokers. The final pathology was pleomorphic carcinoma (*N* = 6), spindle cell carcinoma (*N* = 6), mixed spindle and giant cell carcinoma (*N* = 6), mixed spindle cell/pleomorphic carcinoma (*N* = 2), and giant cell carcinoma (*N* = 1) (Figure 1b). All patients underwent upfront surgery without preoperative chemotherapy or radiation therapy, and none received immunotherapy before or post surgery. Seven patients (33.3%) received adjuvant therapy, including one (4.8%) with radiation therapy and six (28.6%) with chemotherapy. After an average follow‐up of 39 months after surgery, eleven patients (52.4%) had disease recurrence.

**Table 1:** Summary of patient demographic and clinical characteristics.

Characteristics	*N* = 21
Age, years (median)	68 (49–80)
Gender, No.	
Male	14
Female	7
Histological subtype (%)	
Giant	4.8
Pleomorphic	28.6
Spindle	28.6
Mixed	38.1
Tumor size, cm, (median)	4.0 (2.7–8)
Pleural invasion (%)	33.3
Stage (%)	
IA	14.3
IB	33.3
IIA	4.8
IIB	28.6
IIIA	9.5
IIIB	4.8
IV	4.8
Smoking status (%)	
Never	4.8
Ever	95.2
Adjuvant therapy (%)	
Yes	33.3
No	57.1
Unknown	9.5
Recurrence status (%)	
Yes	52.4
No	47.6

### 3.2 Mutational landscape of PSC

The average sequencing depth (WES) was 226x for tumors and 222x for germline controls (uninvolved normal lung tissues) (Supporting Information: Figure S1C,F). A total of 5147 somatic mutations (average 285/tumor, ranging from 5 to 1110, an average of 5.7/Mb) were identified (Supporting Information: Figure S1A, Supporting Information: Table S2). The TMB was not significantly different between the three main histological subtypes of PSC (Supporting Information: Figure S1D). Among those mutations, 3278 were exonic mutations with an average of 182 per tumor (range: 5–728), with mutations of high VAF in CDKN2A and MET (Supporting Information: Figure S1E).

**Figure 2:**
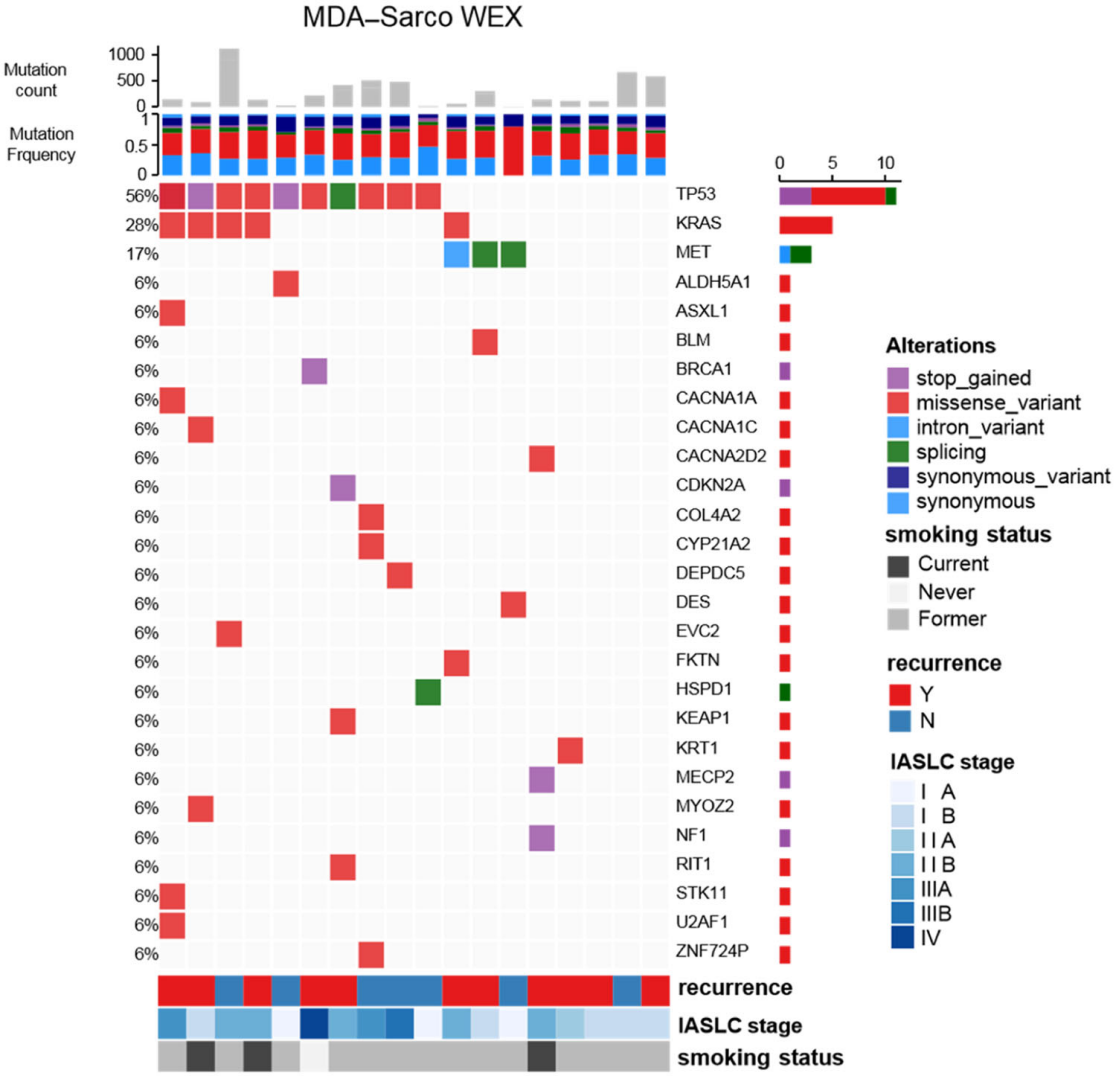
Mutation spectrum across pulmonary sarcomatoid carcinoma (PSC) tumors found using whole‐exome sequencing (WES) across 18 tumors. TP53 is the majority mutation (53%), followed by KRAS in 28% of tumors.

We then examined the pattern of known cancer gene mutations in our cohort, defined as nonsynonymous mutations that lead to pathogenic amino acid changes in oncogenes or truncating mutations in known and previously reported tumor suppressor genes [25, 26] and CancerMine [27]. In total, 27 canonical cancer gene mutations were identified in 15 of 18 tumors with WES data available, validated by deep sequencing of CCP of 409 cancer genes and RNASeq (Figure 2, Supporting Information: Figure S1B) [6]. Among those cancer genes, *TP53* was the most frequently mutated gene (57%; 12/21), followed by *KRAS* (28%; 6/21). The potentially targetable *MET* exon 14‐skipping mutation reported previously [6] was detected in 3 patients in our cohort. None of these three patients carried other cancer gene mutations, such as *TP53* or *KRAS*, supporting a mutual exclusion relationship [28]. Patients harboring *KRAS* mutation were associated with inferior recurrence‐free survival (RFS) and overall survival (OS) in our cohort (*χ* ^2^ test; *p* < 0.015, Supporting Information: Figure S2A,B), consistent with the previous reports in NSCLCs [29, 30, 31]. However, there was no association observed between other cancer gene mutations such as *TP53* mutations and *MET* exon 14‐skipping mutations.

### 3.3 Branching evolution in most PSC tumors

**Figure 3:**
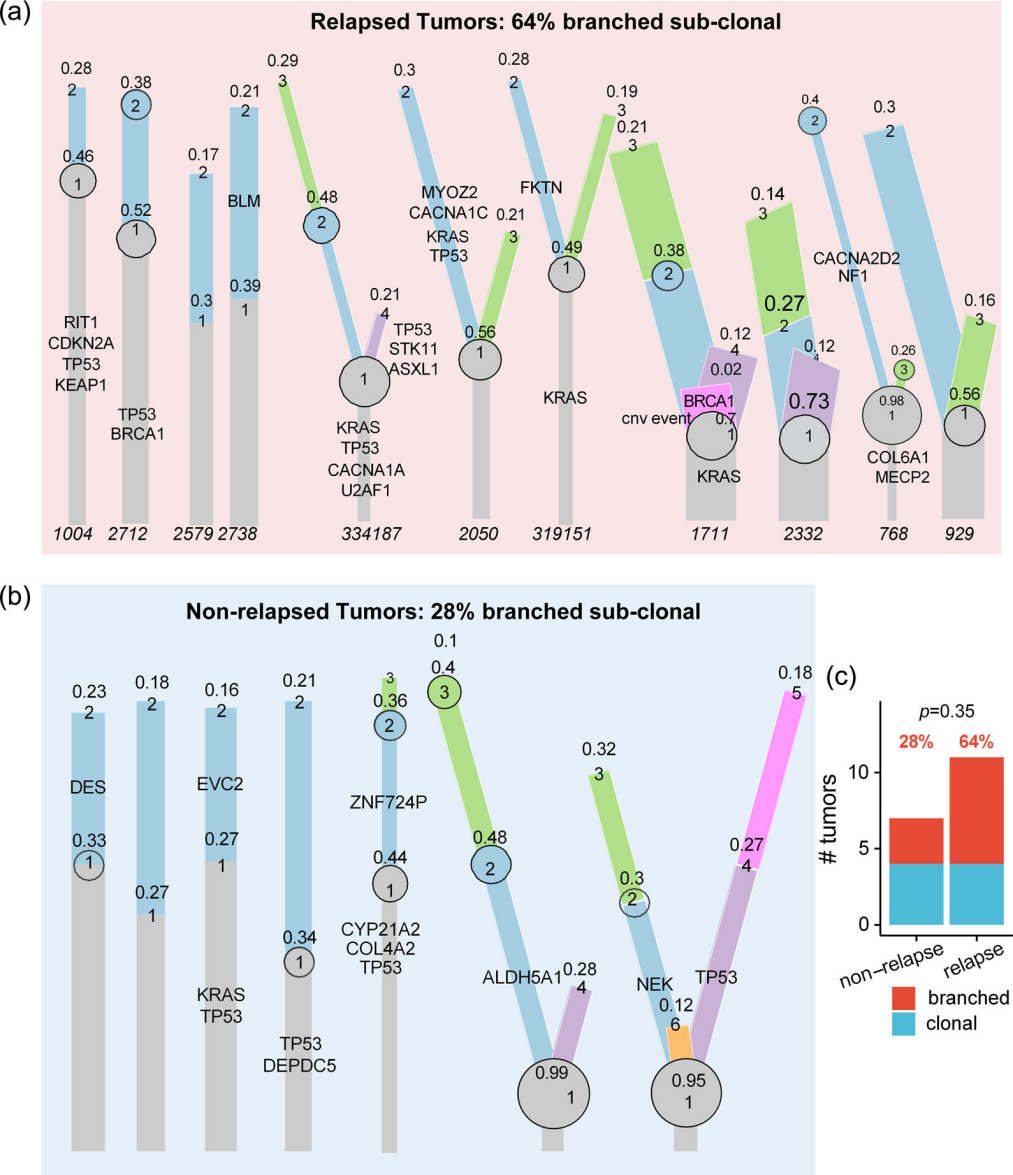
Phylogenetic trees of (a) relapsed tumors and (b) non‐relapsed pulmonary sarcomatoid carcinoma (PSC) tumors derived using mutation and copy‐number data. (c) Relapsed tumors showed an increased number of branches per tumor in 64% of tumors, while branching was detected in 28% of non‐relapsed tumors.

To depict the genomic evolution of these PSC tumors, we used the GATK pooled normal approach followed by TitanCNA and PhyloWGS to estimate the phylogenetic structure of somatic aberrations. Overall, 9 of 18 tumors with available WES data had a branched evolutionary pattern with unique somatic mutations present in two or more subclones. Specifically, 64% of PSC tumors that subsequently relapsed and 28% of nonrelapsed tumors had evidence of a branched evolution (Figure 3a–c). Four of 5 *KRAS* mutations mapped to the trunk (Figure 3a,b), suggesting *KRAS* mutations were early genomic events during the evolution of most PSCs in this cohort. Similar to *KRAS*, 80% of *TP53* mutations were also earlier genomic events mapped to the trunks of the tumors. Interestingly, two distinct *TP53* mutations (a stop‐gain and a missense) were identified in tumor 334187 (Figure 3a), suggestive of convergent evolution, a phenomenon that has been observed in multiple tumors [32, 33, 34].

### 3.4 Gene expression profiles identify two distinct molecular subtypes of PSC

**Figure 4:**
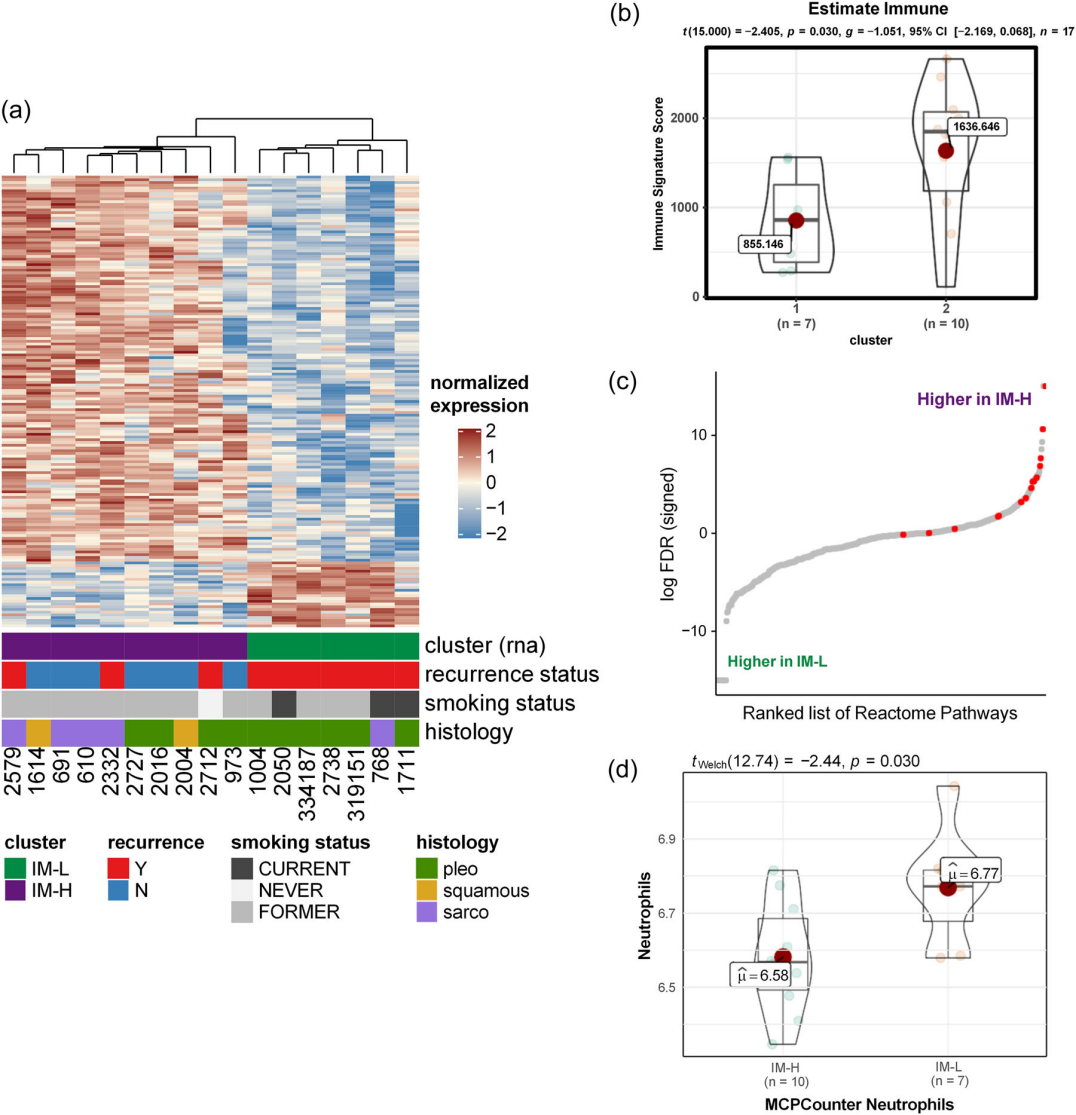
(a) Gene‐expression heatmap of differentially expressed genes across two clusters: Immune High (IM‐H) and Immune Low (IM‐L). (b) Tumor immune infiltration estimated using ESTIMATE indicates higher immune infiltration in IM‐H tumors. (c) Immune‐related reactome pathways (red) are highly expressed in IM‐H tumors. Estimated (d) neutrophils in IM‐H and IM‐L tumors using MCPCounter.

To further understand the molecular landscape of PSCs, we performed RNA sequencing in 17 of the 21 tumors with remaining tumor tissues. Unsupervised clustering using NMF (nonnegative matrix factorization) [35] led to two or six stable clusters (Supporting Information: Figure S3A). Considering the limited sample size, cophenetic distance between the clusters, and silhouette widths of each sample (Supporting Information: Figure S3B), we utilized the two‐cluster system for further analysis. We ranked all protein‐coding genes to differentiate the two subtypes using SAM (significance analysis of microarrays) [36], resulting in 165 candidate genes (*p* < 0.05), among which 24 genes were upregulated in Cluster 1 and 141 enriched in Cluster 2 (Figure 4a, Supporting Information: Table S3). Notably, many of the key pathways represented by the 141 genes upregulated in cluster 2 were related to immune response, such as adaptive immune system, T cell receptor (TCR) signaling, IL‐7 signaling, and GPCR‐related pathways, and so forth (Figure 4c). Conversely, 24 upregulated genes in Cluster 1 belonged to pathways related to metabolism and proliferation, and so forth (Supporting Information: Table S3).

To further understand the genes and pathways driving these two divergent molecular subtypes, we extracted DEG between these two clusters and identified 326 genes upregulated and 360 genes downregulated (*p* < 0.05) in Cluster 2 compared with Cluster 1 (Supporting Information: Figure S3C). We next applied geneset enrichment on a ranked list of genes (Supporting Information: Table S4) based on Reactome, KEGG, and Hallmark genesets to explore upregulated and downregulated pathways in these two subtypes [35, 37, 38]. These analyses demonstrated a significant difference between these two clusters, with most immune‐related pathways (10 of 15) enriched in Cluster 2 (Figure 4c). We then characterized immune infiltration in each tumor using a previously established signature [39], and we observed a significantly higher immune signature in Cluster 2 (hereafter referred to as Immune High, IM‐H) compared with Cluster 1 (hereafter referred to as Immune Low, IM‐L) (Figure 4b, Supporting Information: Table S5). SsGSEA analysis for MSigDB hallmark pathways further revealed that IM‐H tumors were enriched in various immune pathways while IM‐L tumors were enriched in pathways associated with cell proliferation, epithelial–mesenchymal transition (EMT), metabolism, and so forth (Supporting Information: Figures S4 and S5) suggestive of highly aggressive nature and less immune surveillance in IM‐L tumors.

To further understand the immune landscape of these PSCs, we applied ESTIMATE [39] to RNA‐seq data. As expected, IM‐H tumors exhibited a significantly higher immune score and a higher stromal score but a lower tumor purity score (Supporting Information: Figure S6A–C). We further applied MCPCounter [21], CIBERSORT [22], ConsensusTME [23], and xCell [24] to infer the infiltration of different immune cell types in IM‐H versus IM‐L tumors. Cells with consistent trends across at least two methods were used for further analysis with clinical variables. The distinct immune‐centric molecular characteristics between these two clusters were also observed in neutrophils (Figure 4d), B cells (Supporting Information: Figure S6D), endothelial cells (Supporting Information: Figure S6E), plasma cells (Supporting Information: Figure S6F), M1 macrophage fraction (Supporting Information: Figure S6G), and CD4^+^/CD8^+^ T cell ratio [40] (Supporting Information: Figure S6H) further supporting a more active immune repertoire associated with IM‐H PSC tumors. In addition, several tumors in the IM‐H group exhibited higher expression of various immune checkpoint‐related genes, including LAG3, IDO1, and TIGIT (Supporting Information: Figure S7).

### 3.5 Patients with IM‐H versus IM‐L PSC tumors exhibit different survival

**Figure 5:**
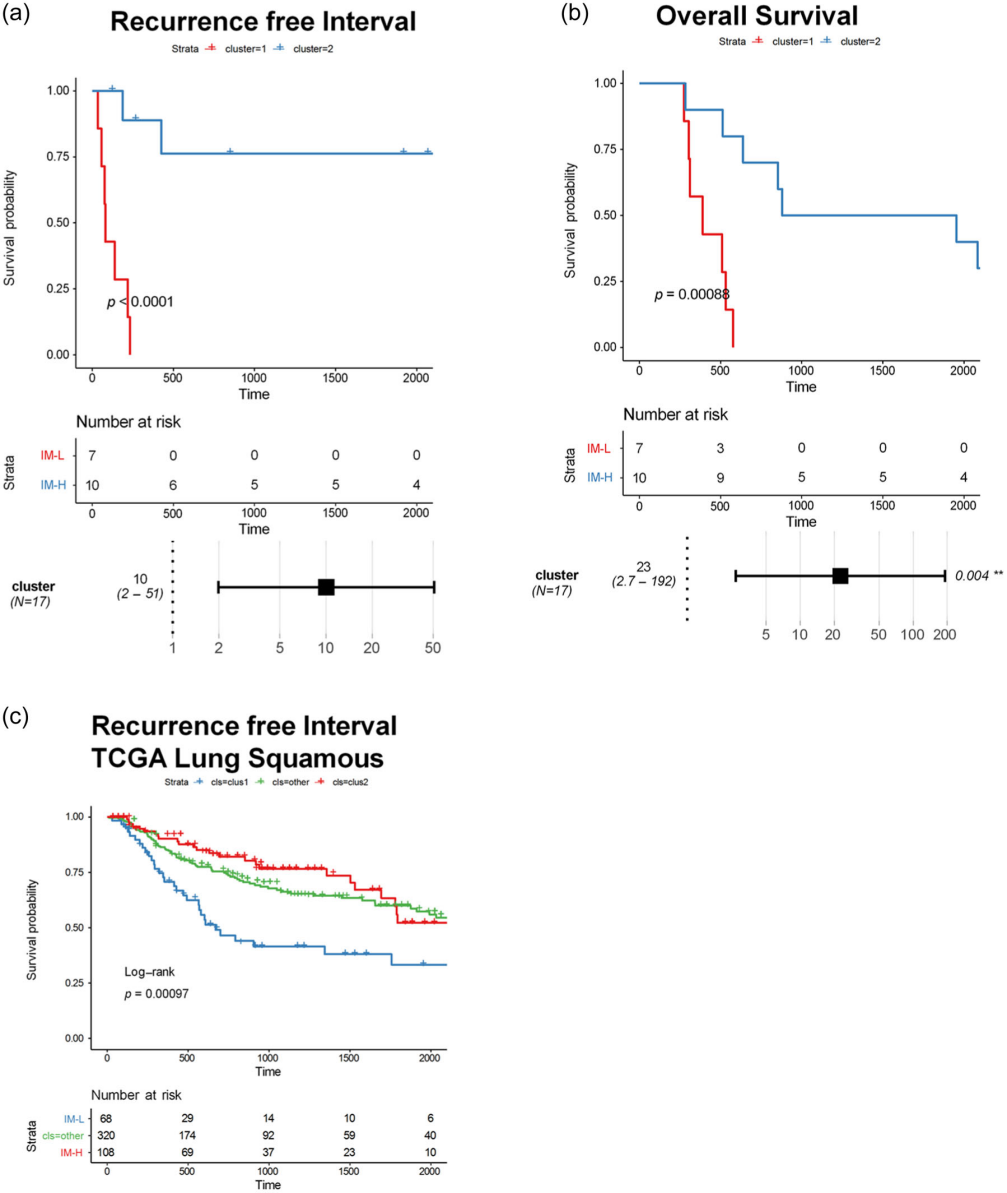
(a) Recurrence‐free survival (RFS) and (b) overall survival (OS) in Immune Low (IM‐L, cluster 1) and Immune High (IM‐H, cluster 2) tumors. (c) Projection of IM‐H and IM‐L subtyping on lung squamous TCGA cohort has prognostic value in nonsarcomatoid lung tumors.

We next sought to assess the potential impact of these molecular subtypes on clinical outcomes of PSC patients. Importantly, all seven patients in the IM‐L cluster have relapsed, compared with only 3 of 10 patients with IM‐H PSC. Furthermore, survival analysis revealed significantly longer RFS and OS in patients clustered into IM‐H group (HR = 10, 95% CI: 2–51, *p* = 0.005 for RFS; HR = 23, 95% CI: 2.7–192, *p* = 0.04 for OS; Figure 5a,b). The associations remained significant in multivariate analysis after adjusting for smoking status, stage, and gender (Supporting Information: Figure S8A,B).

To better understand the correlations of immunogenicity with the clinical outcome, we further investigated the relationship between subtype and patient survival in lung squamous cell carcinoma (LSCC) in the TCGA cohort. We identified 68 patients with IM‐L cluster and 108 patients with IM‐H cluster in LSC (*N* = 320 unassigned), and a favorable prognosis was observed in cluster 2 patients, the same finding with our PSC cohort (Figure 5c). Taken together, these results demonstrated the distinct clinical outcomes in PSC patients with different molecular subtypes, further highlighting the impact of tumor immune infiltration on patient survival.

### 3.6 IM‐L and IM‐H PSC tumors had different genomic features

Finally, we sought to understand the genomic basis underlying the difference in immune infiltration in these PSC tumors. First, we investigated whether the canonical cancer gene mutations that are known to impact immune infiltration and response to ICB [41, 42] would impact the immune infiltration of these PSC tumors. The most commonly mutated cancer genes in this cohort, including TP53, KRAS, and MET, did not seem to associate with different immune infiltration (Supporting Information: Figure S9A–F). Next, we compared IM‐L and IM‐H tumors regarding their TMB, which has been reported to be associated with active immune infiltration and superior benefit from ICB [43, 44]. As shown in Supporting Information: Figure S10, we did not observe a significant difference in TMB between IM‐L and IM‐H tumors, suggesting TMB may not be the main driving force of different immune infiltration of these clusters. Of particular interest, IM‐L tumors demonstrated a trend toward higher CNV burden than IM‐H tumors (Supporting Information: Figure S11), consistent with our previous findings that high CNV burden may be associated with cold immune infiltration [45, 46].

## 4 DISCUSSION

Lung cancer is the leading cause of cancer‐related deaths worldwide. Comprehensive molecular profiling has significantly advanced our understanding of lung cancers, identified novel therapeutic targets, and improved patient survival. However, these efforts have so far focused on its most common subtypes, such as LUAD and LSCC [47, 48, 49]. In contrast to these conventional cancer types, PSC, this exceptionally aggressive lung cancer subtype, exhibited high CD8^+^ T cell density, tumor‐associated macrophages, and PD‐L1 expression and was linked to poorer survival and a higher incidence of postoperative progression [50]. Over the past several years, targetable molecular alterations such as *MET* exon 14 skipping mutations were identified [1, 4, 6, 51, 52, 53, 54, 55, 56]. However, there were very few studies have assessed the comprehensive molecular landscape of PSC using multiomics approaches. A recent study characterized 179 PSCs by immunohistochemistry, next‐generation sequencing, and in silico analysis with respect to clinical, immunological, and molecular features and revealed a high prevalence of MET exon 14 skipping mutations as well as high PD‐L1 expressions in PSCs [56]. In this study, we performed an integrative molecular analysis of 21 PSC samples using targeted gene sequencing, WES and RNA sequencing to comprehensively define the molecular underpinnings of this rare clinical entity. We provided detailed insight into the mutational landscape of PSC and identified two molecular subtypes associated with prognosis. Consistent with previous reports, *TP53* mutations were identified in 57% of cases, and *KRAS* mutations were found to be associated with inferior survival. Unsupervised clustering based on transcriptomic data identified two molecular subtypes of PSC exhibiting high and low immune infiltration. Importantly, IM‐H tumors were associated with favorable RFS and OS, highlighting the importance of tumor immune infiltration in the biological and clinical features of PSCs. PSC represents a therapeutic challenge clinically, with patients often treated with standard chemotherapy and/or radiotherapy while the other NSCLCs provide unsatisfactory success [5]. *MET* exon 14 skipping mutations have provided a new therapeutic target for PSCs, but only in a small proportion of PSC patients, and disease control is often short‐lived for most patients [57].

Immunotherapy by ICB has shown unprecedented durable clinical responses in patients with various cancer types, including NSCLC [58]. ICB has been recently tested in PSCs and demonstrated promising clinical efficacy and tends to be associated with favorable survival [50, 59, 60, 61]. However, the response rate is suboptimal in unselected patient populations [62]. Although ICB is better tolerated than chemotherapy, it does come with severe side effects [63]. As such, establishing reliable biomarkers is urgently needed to identify PSC patients who will most benefit from ICB is critical. A prior study showed that patients with low processing mutations display survival benefits treated with immunotherapy [56]. In this study, we identified two distinct molecular subtypes of PSCs. The IM‐H group was associated with an overall high immune score, high infiltration of immune cell subsets, and high expression of checkpoint molecules that are associated with better response to ICB across different cancer types [64, 65]. Accordingly, patients with IM‐H PSCs could more likely benefit from ICB.

As a retrospective study on rare tumors, our study was limited by the sample size. Therefore, the intriguing findings presented in this study warrant validation in future studies on large cohorts of PSC tumors, which will likely require multi‐institutional collaboration, given the scarcity of resected PSCs. Another caveat is that none of these patients received ICB treatment. Therefore, how well the IM‐H and IM‐L PSCs associate with response to ICB treatment is yet to be determined. Nevertheless, using multiomics approaches, our study provided proof‐for‐principle evidence that gene expression‐based molecular subtyping may be informative for the underlying biology and clinical outcome of patients with PSC, a rare and aggressive lung cancer that is still very poorly understood.

## 5 CONCLUSION

In conclusion, we reported data from integrated genomic and transcriptomic analysis on 21 resected PSC tumors. Twenty‐seven canonical cancer gene mutations were identified, with TP53 the most frequently mutated gene, followed by KRAS. We also identified two distinct molecular subtypes of PSC exhibiting high and low immune infiltration. The IM‐H subtype tumors are associated with favorable clinical outcomes, highlighting the importance of tumor immune infiltration in the biological and clinical features of PSCs. Our study provides evidence that gene expression‐based molecular subtyping may be informative for the underlying biology and clinical outcome of patients with PSC, which is of great translational significance.

## AUTHOR CONTRIBUTIONS

**Sahil Seth**: Conceptualization (equal); formal analysis (equal); investigation (equal); writing—original draft (equal). **Runzhe Chen**: Data curation (equal); formal analysis (equal); investigation (equal); methodology (equal); writing—original draft (equal). **Yang Liu**: Formal analysis (equal); investigation (equal). **Junya Fujimoto**: Investigation (equal). **Lingzhi Hong**: Investigation (equal). **Alexandre Reuben**: Investigation (equal). **Susan Varghese**: Investigation (equal). **Carmen Behrens**: Investigation (equal). **Tina McDowell**: Investigation (equal). **Luisa Solis Soto**: Investigation (equal). **Cara Haymaker**: Investigation (equal). **Annikka Weissferdt**: Investigation (equal). **Neda Kalhor**: Investigation (equal). **Jia Wu**: Investigation (equal). **Xiuning Le**: Investigation (equal). **Natalie I Vokes**: Investigation (equal). **Chao Cheng**: Investigation (equal). **John V. Heymach**: Methodology (equal). **Don L. Gibbons**: Investigation (equal). **P. Andrew Futreal**: Investigation (equal). **Ignacio I. Wistuba**: Investigation (equal); methodology (equal); project administration (equal); supervision (equal). **Humam Kadara**: Investigation (equal); methodology (equal); project administration (equal); supervision (equal); writing—review and editing (equal). **Jianhua Zhang**: Data curation (equal); formal analysis (equal); investigation (equal); methodology (equal). **Cesar Moran**: Investigation (equal); methodology (equal); project administration (equal); supervision (equal); writing—review and editing (equal). **Jianjun Zhang**: Conceptualization (equal); data curation (equal); funding acquisition (equal); investigation (equal); project administration (equal); supervision (equal); writing—review and editing (equal).

## CONFLICT OF INTEREST STATEMENT

Ignacio I. Wistuba reports consulting or advisory roles for AstraZeneca/MedImmune, Bayer, Bristol‐Myers Squibb, Genentech/Roche, GlaxoSmithKline, Guardant Health, HTG Molecular Diagnostics, Merck, MSD Oncology, OncoCyte, Jansen, Novartis, Flame Inc, and Pfizer; has received grants and personal fees from Genentech/Roche, Bristol Myers Squibb, AstraZeneca/MedImmune, HTG Molecular, Merck, and Guardant Health; has received personal fees from GlaxoSmithKline and Oncocyte, Daiichi‐Sankyo, Roche, Astra Zeneca, Pfizer, and Bayer; has received research funding to his institution from 4D Molecular Therapeutics, Adaptimmune, Adaptive Biotechnologies, Akoya Biosciences, Amgen, Bayer, EMD Serono, Genentech, Guardant Health, HTG Molecular Diagnostics, Iovance Biotherapeutics, Johnson & Johnson, Karus Therapeutics, MedImmune, Merck, Novartis, OncoPlex Diagnostics, Pfizer, Takeda, and Novartis. Jianjun Zhang reports research funding from Merck, Johnson and Johnson, and consultant fees from BMS, Johnson and Johnson, AstraZeneca, Geneplus, OrigMed, and Innovent outside the submitted work. John V. Heymach reports honorariums from AstraZeneca, Boehringer‐Ingelheim, Catalyst, Genentech, GlaxoSmithKline, Guardant Health, Foundation Medicine, Hengrui Therapeutics, Eli Lilly, Novartis, Spectrum, EMD Serono, Sanofi, Takeda, Mirati Therapeutics, BMS, BrightPath Biotherapeutics, Janssen Global Services, Nexus Health Systems, EMD Serono, Pneuma Respiratory, Kairos Venture Investments, Roche and Leads Biolabs. Runzhe Chen reports stock options from BeiGene outside the submitted work. Cara Haymaker received funding to institution from Avenge Bio, Sanofi, Dragonfly, BTG, Iovance Biotherapeutics, KSQ, Obsidian and Virogen, personal fees from Regeneron and stock options from BriaCell as a member of the scientific advisory board outside the submitted work. Alexandre Reuben serves on the Scientific Advisory Board and has received honoraria from Adaptive Biotechnologies. Natalie I Vokes reports consulting roles for Sanofi, Oncocyte, Lilly, Regeneron, Amgen, Xencor, Astra Zeneca, Tempus and Pfizer, and travel reimbursement from regeneron. Professor Jianjun Zhang is a member of the *Cancer Innovation* Editorial Board. To minimize bias, he was excluded from all editorial decision‐making related to the acceptance of this article for publication. The remaining authors declare no conflict of interest.

## ETHICS STATEMENT

This study was performed in accordance with the Declaration of Helsinki and was approved by the Institutional Review Board at The University of Texas MD Anderson Cancer Center (Approval Number: PA13‐0589, LAB90‐020, LAB03‐0320).

## INFORMED CONSENT

All patients provided written informed consent.

## Supporting information

Supporting information.

## Data Availability

The data supporting the findings of this study can be obtained from the corresponding author, Jianjun Zhang (jzhang20@mdanderson.org) upon reasonable request.
