# Supplementary material for: Integrative genomic and transcriptomic profiling of pulmonary sarcomatoid carcinoma identifies molecular subtypes associated with distinct immune features and clinical outcomes
Source: Cancer Innov. 2024 Apr 15;3(3):e112. doi: 10.1002/cai2.112 (PMC11212327; doi:10.1002/cai2.112)
Supplement: Supplementary file 1 — Supporting information. [file CAI2-3-e112-s001.docx]

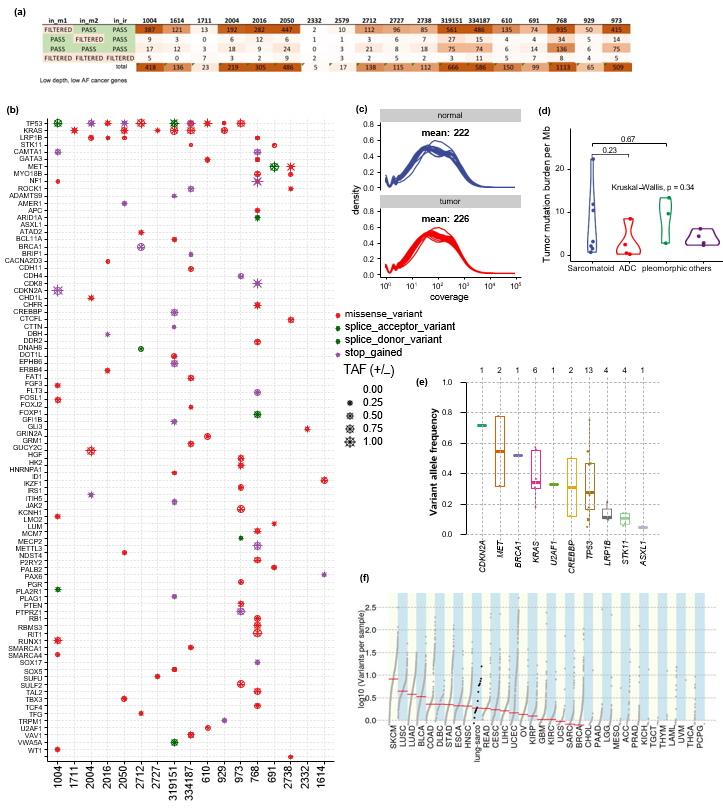


**Figure S1.** (**a)** Number of mutations detected in the cohort using different methods, MuTect (m1), MuTect 2 (m2) and IonReporter (ir), across method specific filters (filtered, PASS). Last row indicated mutations in oncogenes filtered using the three methods but recaputed after manual curation. (**b)** Tumor allele frequence of mutations in the +ve and -ve strand (star and circle) across WES tumors. (**c)** Mean coverage across all exons comparing tumors and adjacent normal tissues. (**d)** Tumor mutation burden (TMB) across three major histopathologic types of tumors in the cohort. (**e)** Variant allele frequency (VAF) of oncogenes/tumor suppressors. (**f)** TMB in this cohort compared across TCGA cohorts.


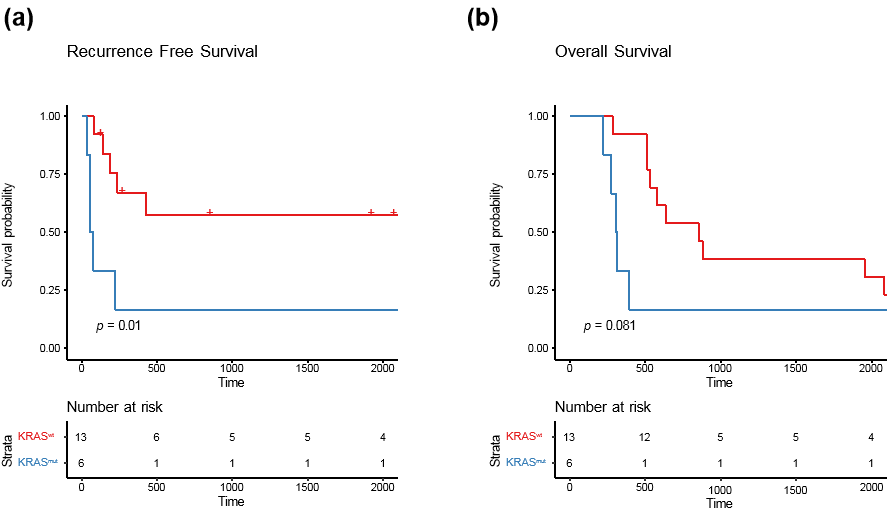


**Figure S2.** (**a)** Recurrence free survival (RFS) and (**b)** overall survival (OS) comparing KRAS mutated tumors and KRAS wild type tumors.


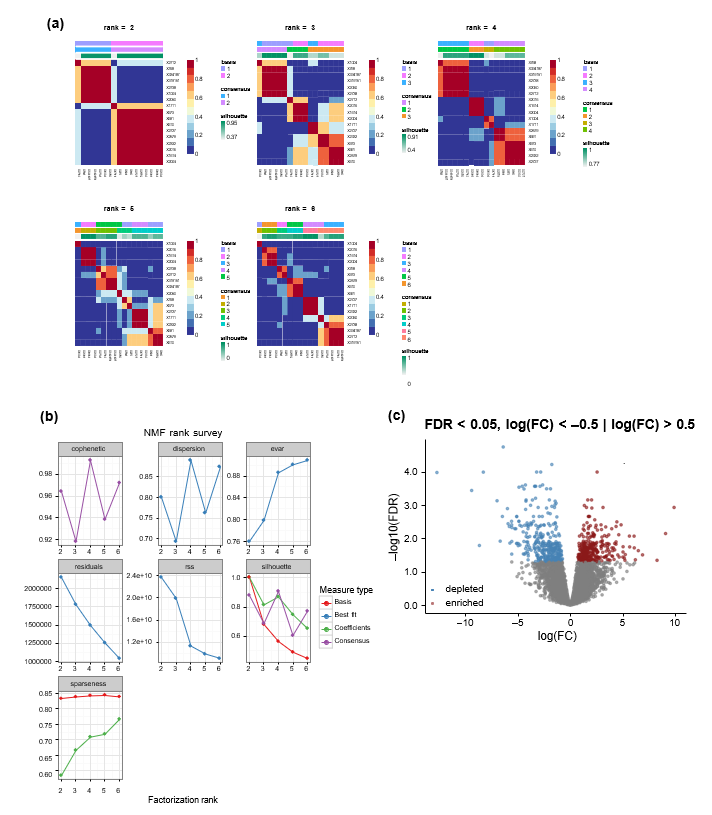


**Figure S3.** (**a)** Non-negative matrix factorization (NMF) comparing rank=2 to rank=6, using various cluster quality metrics like cophenetic distance and ilhouette width. (**b)** Cophenetic distance between the clusters and silhouette widths of each sample suggests N=2 as the optimal cluster size give cohort size and NMF metrics. (**c)** Differentially expressed genes comparing Immune High (IM-H) and Immune Low (IM-L) tumors.


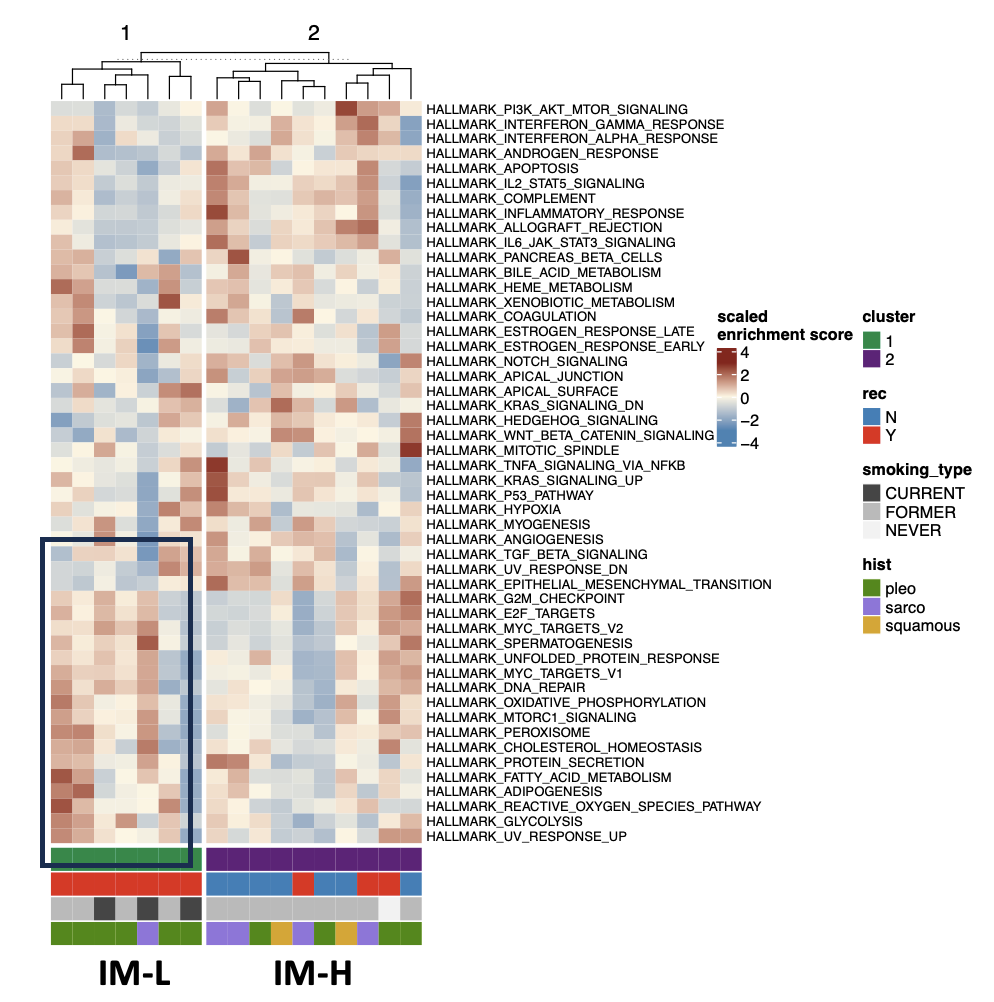


**Figure S4.** ssGSEA for MSigDB hallmark pathways in Immune Low (IM-L) versus Immune High (IM-H) pulmonary sarcomatoid carcinoma (PSC) tumors


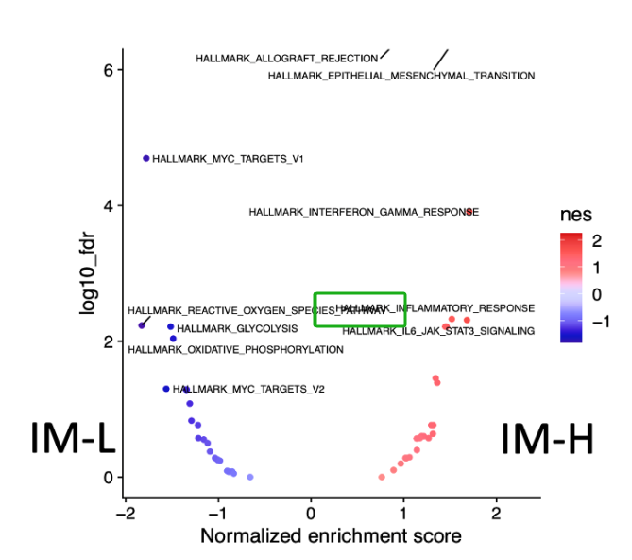


**Figure S5.** Pathways enriched in Immune Low (IM-L) versus Immune High (IM-H) pulmonary sarcomatoid carcinoma (PSC) tumors within MSigDB Hallmark genesets

**Figure S6.** Immune profiles derived from deconvolution of RNA-sequencing data of Immune Low (IM-L) versus Immune High (IM-H) tumors. (**a)** Immune score, (**b)** stromal infiltration score and (**c)** tumor purity using ESTIMATE. Estimated proportions of (**d)** B cells, (**e)** endothelial cells, (**f)** plasma cells, (**g)** M1 macrophages and (**h)** CD4+/CD8+ ratio.


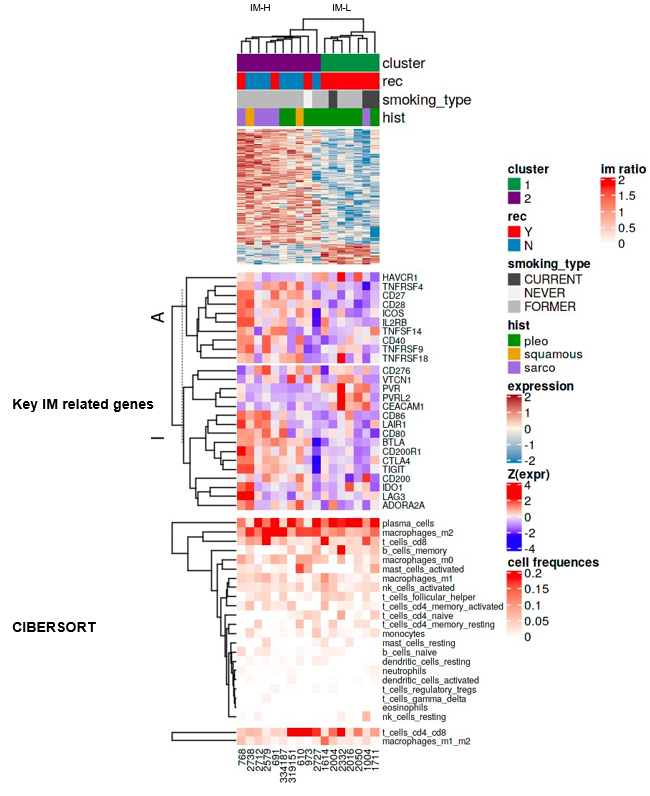


**Figure S7.** Expression of key immune-checkpoint related genes associated with immune inhibitory (I) and immune agonist genes (A) across Immune High (IM-H) and Immune Low (IM-L) tumors, with cell-type proportions.


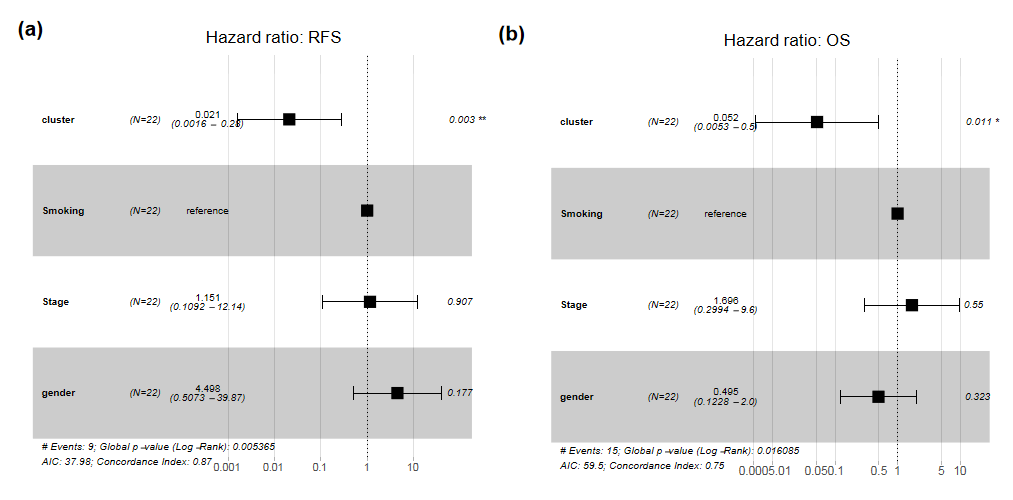


**Figure S8.** Forest plots evaluating the contribution of RNA-cluster, smoking status, tumor stage, and patient gender on (**a)** recurrence free survival (RFS) and (**b)** overall survival (OS).

**Figure S9.** **Comparison of B cells and immune scores derived from deconvolution of** RNA sequencing data between tumors with (1) versus without (0) common cancer gene mutations in this cohort of PSC tumors. (a) KRAS mutant versus KRAS wild type, (b) MET mutant versus MET wild type and c) P53 mutant versus P53 wild type tumors. Estimated immune score between (c) KRAS mutant versus KRAS wild type, (d) MET mutant versus MET wild type and e) P53 mutant versus P53 wild type tumors.

**Figure S10.** Tumor mutation burden (TMB) of Immune Low (IM-L) versus Immune High (IM-H) tumors.

**Figure S11.** Copy number variation (CNV) burden between Immune Low (IM-L) and Immune High (IM-H) tumors.

**Table S1.** Filtered somatic mutations in pulmonary sarcomatoid carcinoma (PSC) tumors

**Table S2.** Somatic mutations identified in pulmonary sarcomatoid carcinoma (PSC) tumors

**Table S3.** Two subtypes (Cluster 1 and 2) of pulmonary sarcomatoid carcinoma (PSC) tumors using SAM (significance analysis of microarrays) by protein-coding genes

**Table S4.** Geneset-enrichment on a ranked list of genes based on Reactome, KEGG and Hallmark genesets in Cluster 1 and 2 pulmonary sarcomatoid carcinoma (PSC) tumors

**Table S5.** Estimated immune infiltration in Cluster 1 and 2 pulmonary sarcomatoid carcinoma (PSC) tumors
